# Litter Mixing Alters Microbial Decomposer Community to Accelerate Tomato Root Litter Decomposition

**DOI:** 10.1128/spectrum.00186-22

**Published:** 2022-05-23

**Authors:** Xue Jin, Zhilin Wang, Fengzhi Wu, Xiaogang Li, Xingang Zhou

**Affiliations:** a Key Laboratory of Biology and Genetic Improvement of Horticultural Crops (Northeast Region), Ministry of Agriculture and Rural Affairs, Northeast Agricultural University, Harbin, China; b Department of Horticulture, Northeast Agricultural University, Harbin, China; c College of Biology and the Environment, Nanjing Forestry University, Nanjing, China; University of Southern Denmark

**Keywords:** litterbags, litter decomposition, litter mixing, microbial community, nonadditive effects

## Abstract

Mixing plant litters of multiple species can alter litter decomposition, a key driver of carbon and nutrient cycling in terrestrial ecosystems. Changes in microbial decomposer communities is proposed as one of the mechanisms explaining this litter-mixture effect, but the underlying mechanism is unclear. In a microcosm litterbag experiment, we found that, at the early stage of decomposition, litter mixing promoted tomato root litter decomposition, thus generating a synergistic nonadditive litter-mixture effect. The transplanting decomposer community experiment showed that changes in microbial decomposer communities contributed to the nonadditive litter-mixture effect on tomato root litter decomposition. Moreover, litter mixing altered the abundance and diversity of bacterial and fungal communities on tomato root litter. Litter mixing also stimulated several putative keystone operational taxonomic units (OTUs) in the microbial correlation network, such as Fusarium sp. fOTU761 and Microbacterium sp. bOTU6632. Then, we isolated and cultured representative isolates of these two taxa, named Fusarium sp. F13 and Microbacterium sp. B26. Subsequent *in vitro* tests found that F13, but not B26, had strong decomposing ability; moreover, these two isolates developed synergistic interaction, thus promoted litter decomposition in coculture. Addition of F13 or B26 both promoted the decomposing activity of the resident decomposer community on tomato root litter, confirming their importance for litter decomposition. Overall, litter mixing promoted tomato root litter decomposition through altering microbial decomposers, especially through stimulating certain putative keystone taxa.

**IMPORTANCE** Microbial decomposer community plays a key role in litter decomposition, which is an important regulator of soil carbon and nutrient cycling. Though changes in decomposer communities has been proposed as one of the potential underlying mechanisms driving the litter-mixture effects, direct evidence is still lacking. Here, we demonstrated that litter mixing stimulated litter decomposition through altering microbial decomposers at the early stage of decomposition. Moreover, certain putative keystone taxa stimulated by litter mixing contributed to the nonadditive litter-mixture effect. *In vitro* culturing validated the role of these taxa in litter decomposition. This study also highlights the possibility of regulating litter decomposition through manipulating certain microbial taxa.

## INTRODUCTION

Plant litter decomposition is a key regulator of carbon and nutrient cycling, thereby is crucial to maintain several ecosystem functioning such as soil fertility and plant productivity (1, 2). The rate of litter decomposition is regulated by various interacting factors, including litter quality (e.g., physical and chemical characteristics of litter), environmental conditions (e.g., temperature and moisture) and the decomposer community (e.g., bacteria and fungi, detritivore fauna) (3–5). Litter from different plant species usually mix and decompose together rather than alone. For example, agricultural practices such as intercropping, crop rotation, cover cropping, and organic amendment can result in litter mixtures of different crop species (6, 7). Litter mixtures can decompose at different rates than would be predicted from the component species, resulting either faster (synergistic effect) or slower (antagonistic effect) decomposition rates. This phenomenon is known as the nonadditive litter-mixture effect on litter decomposition (1, 8, 9).

The nonadditive litter-mixture effect is a consequence of complex interactions between litter species mediated by abiotic factors and decomposer communities (10). Several nonexclusive mechanisms have been proposed to explain the nonadditive litter-mixture effect (1), such as (i) transfer of nutrients and inhibitory compounds: nutrients (e.g., nitrogen) transferred by leaching or fungal hyphae from nutrient-rich to nutrient-poor litter may enhance the decomposition rate of nutrient-poor litter, while transfer of inhibitory compounds (e.g., tannins and polyphenols) can results in an antagonistic litter-mixture effect (11, 12); (ii) modification of microclimatic conditions: plant litter species, whose physical characteristics improve the microclimatic conditions (e.g., moister) for decomposers, can stimulate the decomposition of their co-occurring litter species (13); and (iii) changes in decomposer communities: changes in the habitat and resource in the litter mixture can alter the composition, diversity and function of decomposer communities, and thus alter decomposition (1). For example, the magnitude and direction of litter-mixture effects have been shown to be dependent on the presence and identity of detritivore fauna (14–16).

Microorganisms, as an important driver of litter decomposition, are sensitive to the condition of the environment they inhabit, such as the quality and quantity of the litter, temperature, and moisture (1, 5). Moreover, detritivore fauna can affect the microbial community directly through grazing or indirectly through altering the microclimate (14, 15). Recent studies have demonstrated that litter mixing can alter the abundance and diversity of microbial communities on the litter (17, 18). However, there is evidence that differences in the microbial decomposer communities often but not always lead to changes in litter decomposition rate (17, 19–21). This is because functional redundancy may occur in microbial communities—in other words, a change in community composition not necessarily produce a change in ecosystem processes regulated by this community (22–24). Therefore, though it is intuitive to speculate that litter mixing can affect litter decomposition through altering microbial decomposer communities, implicit evidence for this hypothesis is still lacking.

Microorganisms exist with complex interrelationships among the myriad of members of the community, and interspecific interactions are essential for community assembly and ecosystem functioning (25–30). Several mathematical methods (e.g., co-occurrence network analysis) have been developed to infer potential interactions among microorganisms in a community (31). Microbial keystone taxa are highly connected taxa that exert a considerable influence on the assembly and functioning of a community (32). The effects of abiotic and biotic environmental factors on the microbial community can be mediated via keystone taxa (32–34). An efficient decomposition of plant litter requires the complex interactions among members of the microbial community (2, 35, 36). Nonrandom co-occurrence patterns of microbial decomposer community have been observed, and microbial taxa with high decomposing ability (e.g., Fusarium sp.) were potential keystone taxa (3, 5, 37, 38). However, the role of putative keystone taxa in mediating the nonadditive litter-mixture effect is still unclear.

In this study, using litter of six plant species [i.e., tomato (Solanum lycopersicum), cucumber (Cucumis sativus L.), eggplant (Solanum melongena L.), maize (Zea mays), wheat (Triticum aestivum), and wild rocket (*Diplotaxis tenuifolia*)], we tested whether changes in microbial decomposer communities were responsible for the nonadditive litter-mixture effect. We used tomato litter as focal litter. Root litter of eggplant and cucumber were used because eggplant and cucumber were usually rotated with tomato in agricultural production, while aboveground and belowground materials of maize, wheat and wild rocket were used because the whole plants were incorporated into the soil when these crops were used as cover crops (7, 39). First, in microcosm experiments with litterbags containing litter of six plant species alone and mixtures of two, four and six-species, we evaluated the effect of litter mixing on litter decomposition and a component litter, tomato root litter. Second, we evaluated the role of microbial communities in mediating the nonadditive litter-mixture effect on the decomposition of tomato root litter. Third, we characterized the abundance and diversity of microbial communities on tomato root litter and performed network analysis. Finally, putative keystone taxa were subsequently isolated and characterized. We hypothesized that (i) litter mixing would alter the assembly of microbial decomposer communities, which could exert functional consequences for litter decomposition; and (ii) putative keystone taxa would be an important mediator of the relationship between the litter-mixture effect and microbial decomposer communities.

## RESULTS

### Litter-mixture effect on litter mass loss.

**FIG 1:**
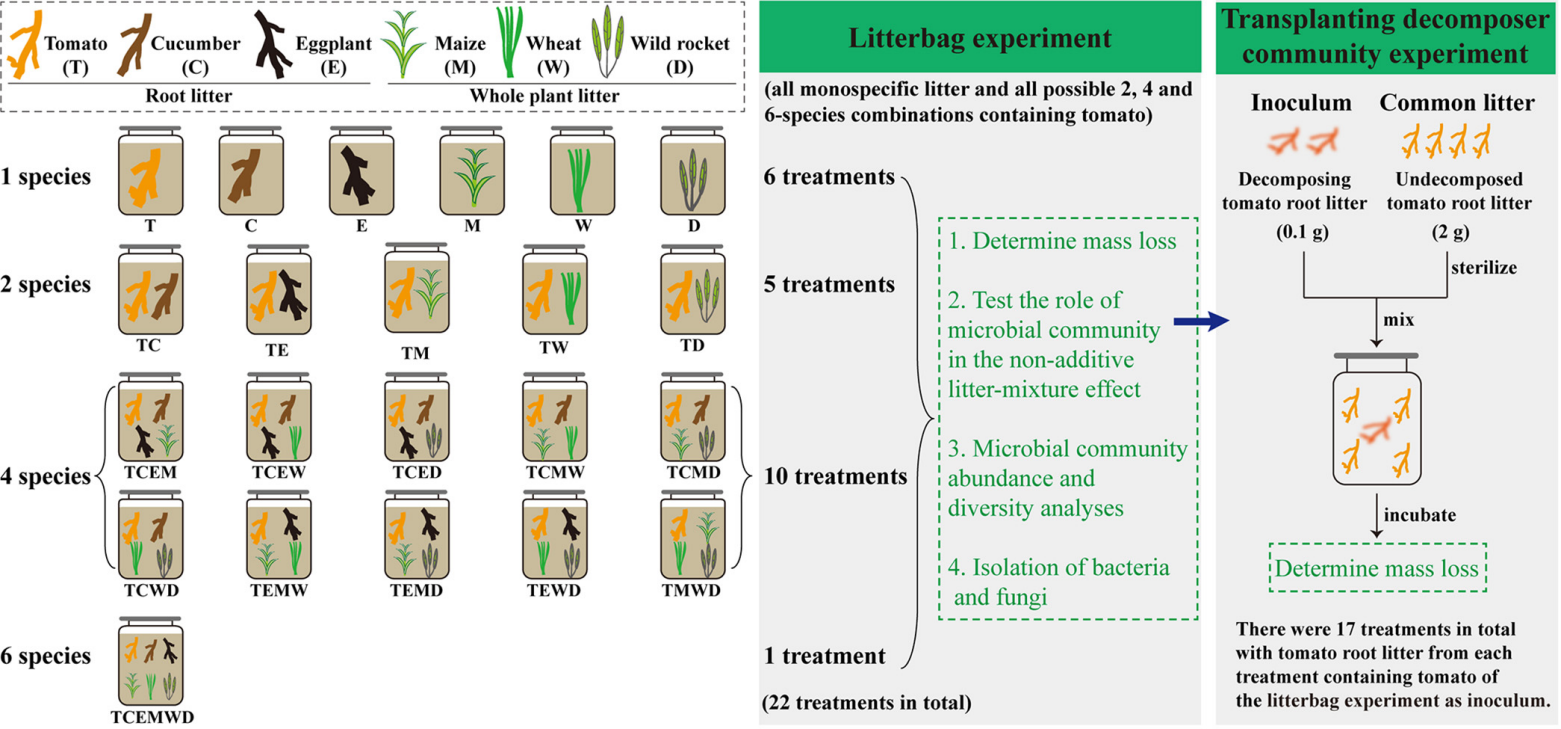
The experimental design. Litter of six plant species were used in this study. For the litterbag experiment, monospecific litter of each six species and all the possible two, four and six-way combinations of tomato with the other five species were included. All treatments were used for determining mass loss, while treatments containing tomato were used to analyze microbial community abundance and diversity, isolate bacteria and fungi. In the transplanting decomposer community experiment, decomposed tomato root litter from the litterbag experiment were used as inoculum to test the function of decomposer communities.

**FIG 2:**
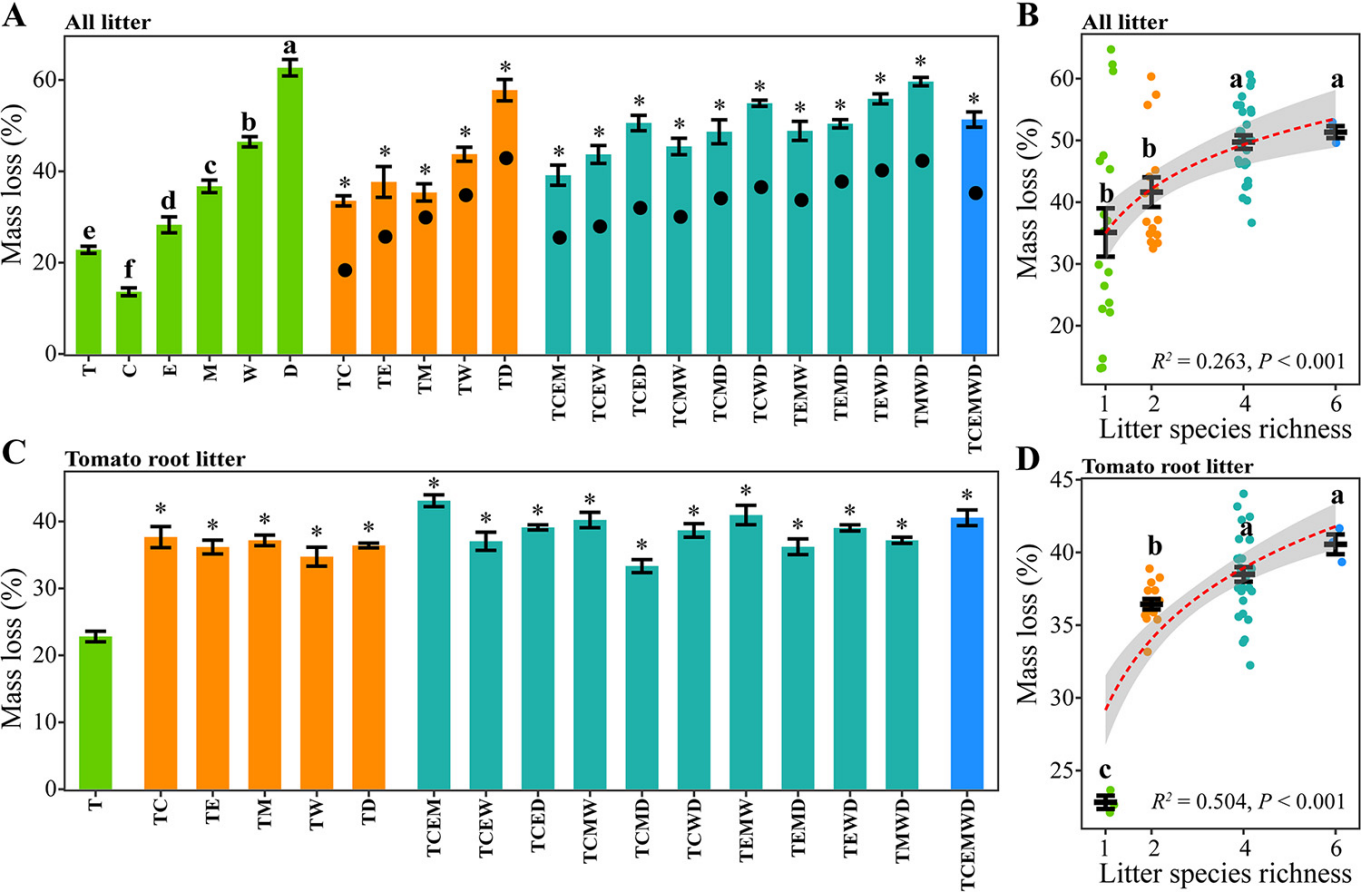
Litter mass loss in the litterbag experiment. (A) Mass loss of all litter for each treatment. For monospecific treatments, different letters indicate significant differences (Tukey's HSD test, *P < *0.05). Black dots indicate the expected mass loss (the mean mass loss of the component litter species in isolation). * indicate significant difference between the observed and the expected mass loss (Student's *t* test, *P < *0.05). (B) Effects of litter species richness on mass loss of all litter. (C) Tomato root litter mass loss for each treatment. * indicate significant different with the monospecific treatment (Student's *t* test, *P < *0.05). (D) Effects of litter species richness on tomato root litter mass loss. Different letters indicate significant differences (Tukey's HSD test, *P < *0.05). For (A) and (C), values are represented as mean ± SE (*n *=* *3). For (B) and (D), dashed red lines show the linear or log-linear regression fittings and shaded areas represent 95% confidence intervals. T, tomato; C, cucumber; E, eggplant; M, maize; W, wheat; D, wild rocket.

The separation of component species behavior within the litter mixture is a prerequisite to identify the mechanisms by which litter mixing influences decomposition (14). Thus, we used the two-compartment litterbag method here (40) (Fig. 1, Fig. S1 in the supplemental materials; see Materials and Methods for details). Generally, litter of maize, wheat and wild rocket had higher nitrogen but lower lignin contents, and decomposed faster than that of litter of tomato, cucumber and eggplant (Tukey’s HSD test, *P < *0.05) (Fig. 2A, Table S1). Most litter mixtures decomposed faster than predicted (Student's *t* test, *P < *0.05) (Fig. 2A). Both litter species composition and richness altered litter mass loss [Two-way analysis of variance (ANOVA), *P < *0.001]. Litter mass loss increased with increasing litter species richness (*P < *0.01) (Fig. 2B). The presence of wheat and wild rocket enhanced decomposition of the litter mixture (*P < *0.01) (Fig. S2A).

Tomato root litter mass loss was altered by both litter species composition and richness (Two-way ANOVA, *P < *0.01). Tomato root litter mass loss was higher in all mixtures than in the monospecific treatment (Student's *t* test, *P < *0.01) (Fig. 2C), and showed an overall increase with litter species richness (*P < *0.01) (Fig. 2D). The presence of cucumber, eggplant, maize and wheat promoted tomato root litter decomposition (*P < *0.05) (Fig. S2A).

### Decomposing ability of microbial communities on tomato root litter.

**FIG 3:**
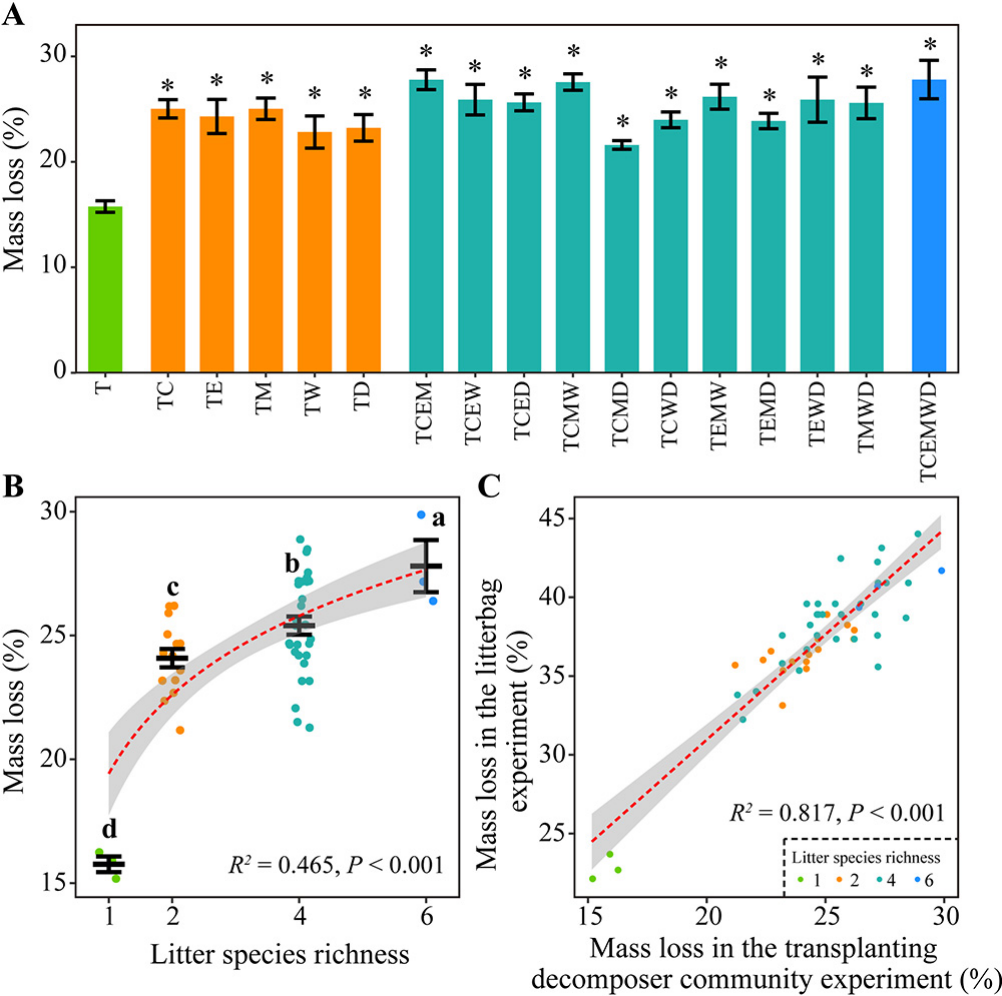
Tomato root litter mass loss in the transplanting decomposer community experiment. (A) Tomato root litter mass loss for each treatment. * indicate significant different with the monospecific treatment (Student's *t* test, *P < *0.05). Values are represented as mean ± SE (*n *=* *3). (B) Effects of litter species richness on the mass loss of tomato root litter. Different letters indicate significant differences (Tukey's HSD test, *P < *0.05). (C) Relationship between tomato root litter mass loss in the double-compartment litterbag experiment and that in the transplanting decomposer community experiment. Dashed red lines show the linear or log-linear regression fittings and shaded areas represent 95% confidence intervals. T, tomato; C, cucumber; E, eggplant; M, maize; W, wheat; D, wild rocket.

A transplanting decomposer community experiment was used to evaluate the decomposing ability of microbial communities on tomato root litter from the litterbag experiment (Fig. 1; see Materials and Methods for details). We found that decomposer inocula of all mixtures from the litterbag experiment had a higher decomposing ability than that of the monospecific tomato root litter treatment (Student's *t* test, *P < *0.01) (Fig. 3A). The ability of these inocula to decompose tomato root litter increased linearly with litter species richness (*P < *0.001) (Fig. 3B). Moreover, the mass loss rate of tomato root litter in the transplanting decomposer community experiment was positively relative to that in the litterbag experiment (*P < *0.001) (Fig. 3C).

### Litter-mixture effect on microbial abundances and diversities.

**FIG 4:**
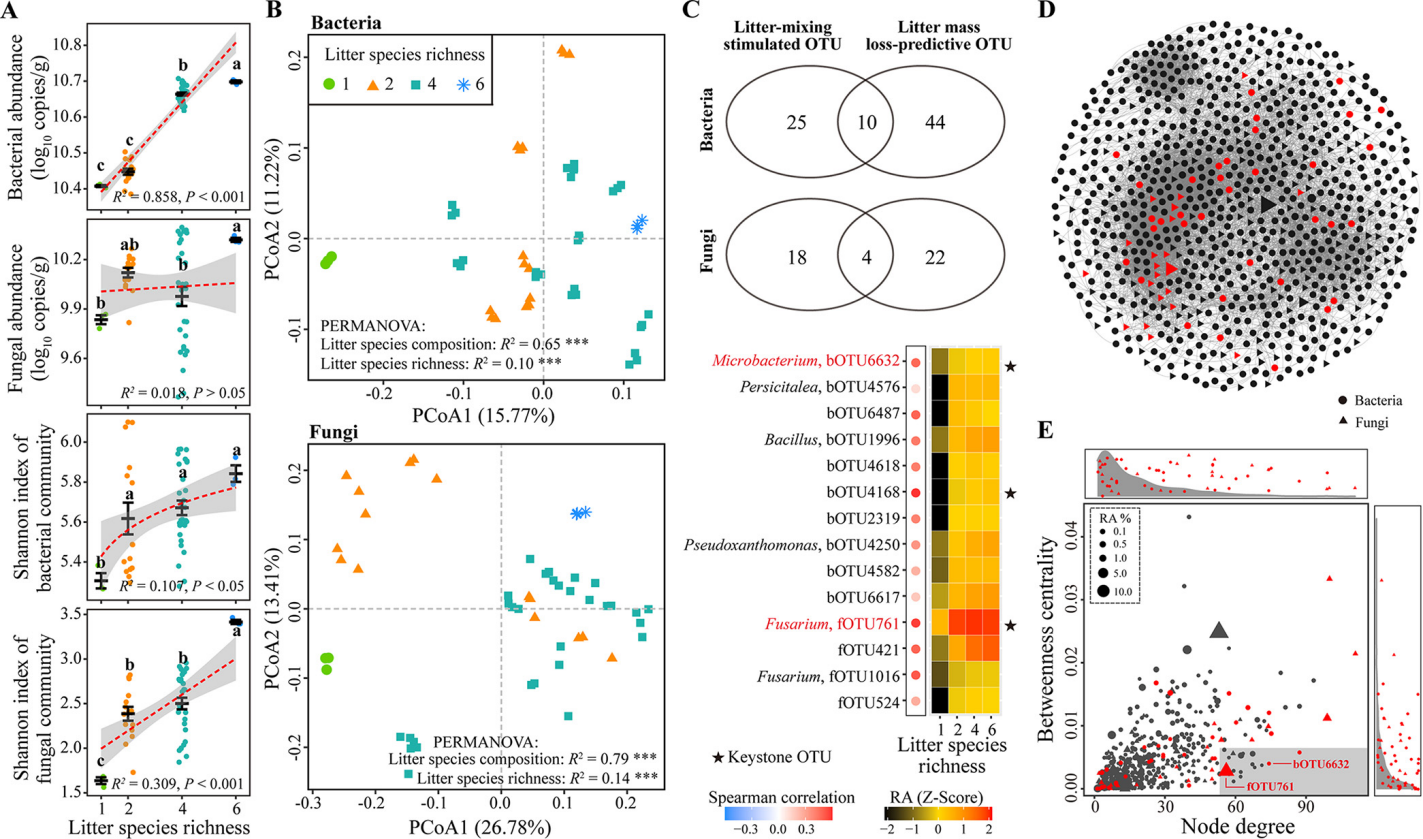
The abundance and diversity of microbial communities on tomato root litter. (A) Effects of litter species richness on abundances and Shannon index of microbial communities. Different letters indicate significant differences (Tukey's HSD test, *P < *0.05). Dashed red lines show the linear or log-linear regression fittings and shaded areas represent 95% confidence intervals. (B) The β-diversities of microbial communities. *** indicates *P < *0.001. (C) Microbial OTUs that were both stimulated by litter mixing and belonged to top-ranking mass loss-predicative OTUs. Venn plots show the numbers of shared and unique OTUs that were stimulated by litter mixing and belonged to top-ranking mass loss-predicative OTUs. The heatmap shows the relative abundances of OTUs that were both stimulated by litter mixing and belonged to top-ranking mass loss-predicative OTUs. The bubbles on the left panel show the Spearman’s correlations between the relative abundance of each OTU and tomato root litter mass loss. (D) The co-occurrence network showing significant correlations (ρ > 0.6, BH-corrected *P < *0.01) between OTUs. The size of each node is proportional to the relative abundance of the OTU. (E) Degree-betweenness centrality plot of OTUs in the network. Keystone OTUs have gray background. Side panels show the distributions of node degrees and betweenness centrality for OTUs stimulated by litter mixing compared to the density of all OTUs in the network. For (D) and (E), OTUs stimulated by litter mixing are in red color.

Real-time PCR and amplicon sequencing were performed to analyze bacterial and fungal communities on tomato root litter in the litterbag experiment. Litter species richness and composition altered the abundances and α-diversities of both bacterial and fungal communities on tomato root litter (ANOVA, *P < *0.001). Bacterial abundance, Shannon indices of both bacterial and fungal communities increased linearly or log-linearly with increasing litter species richness (*P < *0.05) (Fig. 4A). Linear regression analyses found that bacterial abundance, bacterial and fungal Shannon indices were positively related to the mass loss of tomato root litter (*P < *0.05) (Fig. S3 in the supplemental material). The presence of all other litter species stimulated bacterial abundance, while the presence of cucumber stimulated fungal abundance (*P < *0.05) (Fig. S2B). The presence of cucumber and wheat promoted bacterial Shannon index, while the presence of eggplant, maize, wheat and wild rocket promoted fungal Shannon index (*P < *0.05) (Fig. S2C).

For both bacterial and fungal communities, the monospecific treatment was clearly separated from the litter mixtures on the principal coordinates analysis (PCoA) plots (Fig. 4B). Permutational multivariate analysis of variance (PERMANOVA) showed litter species richness and composition altered both bacterial and fungal community β-diversities (*P < *0.001) (Fig. 4B).

### Litter-mixing sensitive and litter mass loss-predictive taxa.

Litter mixing altered the relative abundances of several dominant bacterial phyla and fungal orders (Fig. S4A). For example, increasing litter species richness increased the relative abundances of bacterial phyla/class Deltaproteobacteria and Verrucomicrobia and fungal order Russulales, while decreased that of bacterial class Gammaproteobacteria (*P < *0.01) (Fig. S4B). As identified with both indicator species analysis and likelihood ratio test, 35 bacterial and 22 fungal OTUs had higher relative abundances in litter mixtures than in the monospecific treatment (Fig. S4C). Most of these bacterial and fungal OTUs stimulated by litter mixing belonged to bacterial phylum Bacteroidetes and fungal order Hypocreales respectively.

Regression Random Forest models were established to predict important microbial OTUs mediating tomato root litter decomposition. The models explained 65.02% and 77.73% of the variance related to litter mass loss rate for bacterial and fungal communities, respectively. Tenfold cross-validation further identified 54 and 16 top-ranking mass loss-predicative bacterial and fungal OTUs, respectively (Fig. S5). These top-ranking bacterial OTUs mainly belonged to Proteobacteria and Bacteroidetes, while fungal OTUs mainly belonged to Hypocreales and Sordariales. Meanwhile, several of these top-ranking mass loss-predicative OTUs (10 bacterial OTUs and four fungal OTUs) were stimulated by litter mixing (Fig. 4C).

### Co-occurrence networks and putative keystone taxa.

A co-occurrence network containing both bacterial and fungal OTUs was constructed (Fig. 4D). The modularity values of the co-occurrence networks were higher than 0.4 (Table S2 in the supplemental material). Compared with the Erdös-Réyni random networks, empirical networks had greater values of average path length, average clustering coefficient and modularity. Most OTUs stimulated by litter mixing were included in the co-occurrence network (98%), and generally had low to medium node degree and betweenness centrality values (Fig. 4E). In total, 19 OTUs were identified as keystone OTUs, which had high node degree and low betweenness centrality values. It's worth noting that two keystone OTUs with known phylogenetic information (i.e., *Microbacterium* sp. bOTU6632 and Fusarium sp. fOTU761) belonged to top litter mass loss-predictive OTUs and were stimulated by litter mixing (Fig. 4C and E).

### Experimental testing of putative keystone taxa.

**FIG 5:**
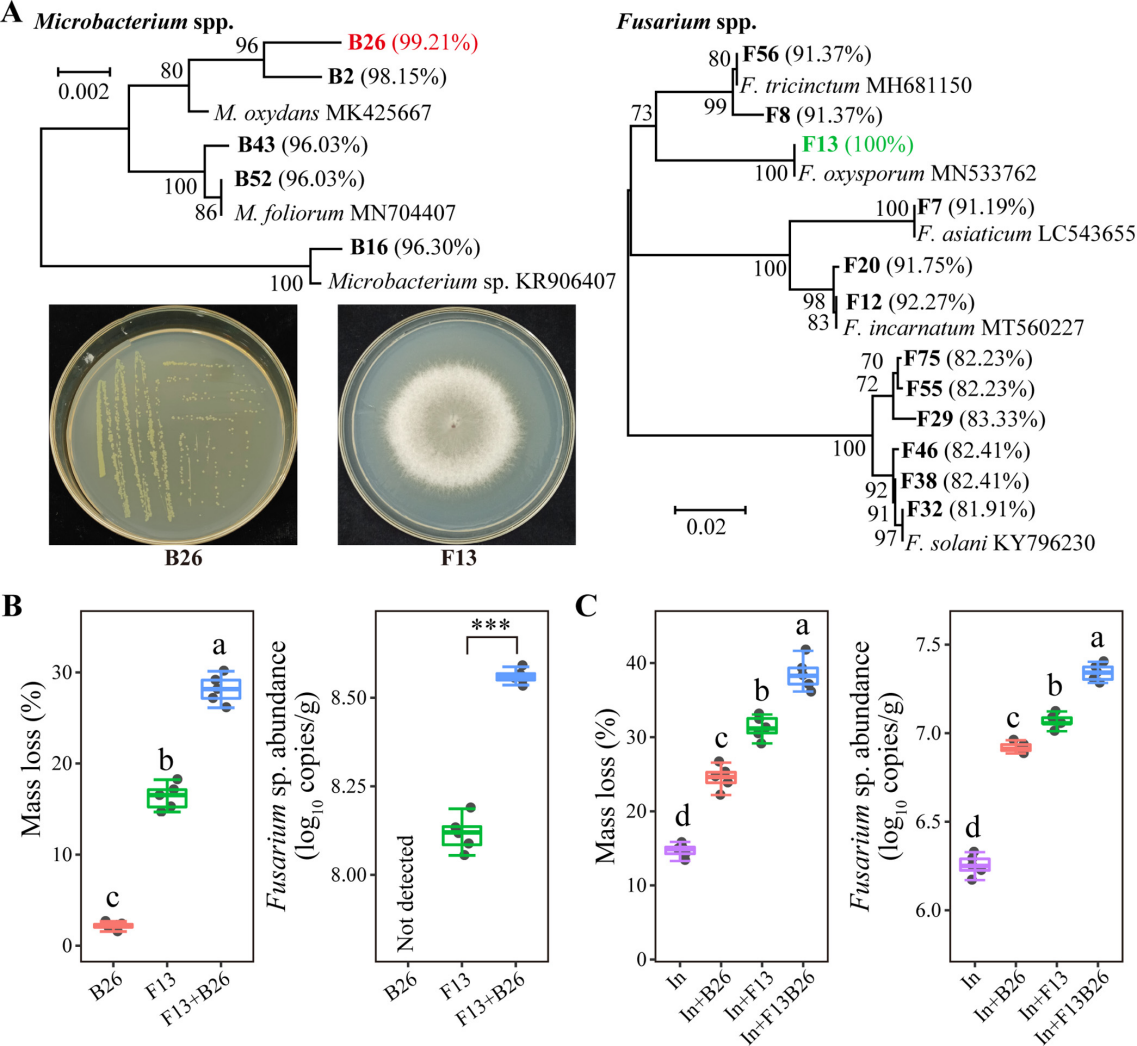
Isolated *Microbacterium* and Fusarium spp. and their decomposing abilities. (A) The neighbor-joining trees showing the phylogenetic relationships of isolated *Microbacterium* and Fusarium spp. Isolates from this study are in bold letters. Reference strains from the NCBI database with their accession numbers are in regular letters. Numbers in parentheses are the sequence similarities of each *Microbacterium* and Fusarium spp. strain with bOTU6632 or fOTU761, respectively. Bootstrap values are based on 1,000 resampling and shown at the branching points. The photographs show the colony morphologies of B26 and F13 grown on Luria-Bertani agar and potato dextrose agar, respectively. (B) The abilities of B26 and F13 in isolate, and their mixture to decompose autoclaved tomato root litter. (C) Effects of addition of B26 and F13 on the decomposing ability of resident microbial community on tomato root litter and Fusarium sp. abundance Decomposing tomato root litter as an inoculant of resident decomposing community (In). Different letters indicate significant differences (Tukey's HSD test, *P < *0.05).

We attempted to isolate Microbacterium and Fusarium spp. to test their role in litter decomposition. In total, we isolated 11 Microbacterium sp. and 59 Fusarium sp. isolates from tomato root litter in the litterbag experiment. After elimination of potential clonal duplicates, i.e., isolates with 100% identity of the 16S rRNA gene or ITS sequences (28), we obtained five Microbacterium sp. and 12 Fusarium sp. isolates. Further, we selected Microbacterium sp. B26 and Fusarium sp. F13 because they displayed the highest sequence similarity with bOTU6632 (99.21%) and fOTU761 (100%), respectively, among these isolates (Fig. 5A).

Then, we assessed the ability of these two isolates to decompose autoclaved tomato root litter. F13 but not B26 in isolation, had a relatively strong litter decomposing ability (Fig. 5B). Meanwhile, the mixture of B26 and F13 resulted in a synergistic effect on decomposing tomato root litter (independent-samples Student's *t* test, *P < *0.05). Real-time PCR analysis found the treatment inoculated with both B26 and F13 had a higher abundance of Fusarium sp. than the treatment inoculated with F13 alone (*P < *0.05) (Fig. 5B).

Finally, we evaluated the effects of B26 and F13 on the decomposing ability of the resident microbial decomposer community on tomato root litter. B26 and F13 in isolate, and the mixture of B26 and F13 promoted the decomposing ability of the microbial inoculum from decomposing tomato root litter and Fusarium sp. abundance on tomato root litter (Tukey’s HSD test, *P < *0.05) (Fig. 5C). Moreover, the mixture of B26 and F13 had a higher stimulating effect than B26 and F13 in isolate (*P < *0.05).

## DISCUSSION

### Litter mixing promoted litter decomposition and altered the assembly of microbial decomposer communities.

We found that litter mixing accelerated the mass loss rates of the whole litter mixture and tomato root litter, which validated the finding that synergistic nonadditive effects were more prevalent than antagonistic nonadditive effects (8, 9). As with several previous litter-mixing studies (18, 41), litter mixing altered the assembly of bacterial and fungal communities on tomato root litter. Moreover, litter species identity exerted significant effects on microbial abundance and α-diversity. Specifically, the presence of certain litter species with both high and low quality increased microbial abundance and α-diversity, indicating that both increasing in resource availability and habitat heterogeneity might be responsible for this observed synergistic effects on microbial abundance and α-diversity (18, 42). Litter chemical composition and diversity have been shown to be important functional traits explaining the litter-mixture effect on belowground ecosystem function (42). Further studies relating the chemical composition of the litter mixture to microbial decomposer community assembly can help to gain a better understanding of the mechanism underlying the litter-mixture effect.

### Changes in microbial decomposer communities were linked to the nonadditive litter-mixture effect.

The high diversity and adaptive ability of microbial communities may confer functional redundancy across different microbial communities (22, 23, 43). Thus, changes in the abundance and diversity of microbial decomposer communities do not warrant a direct causal relationship between these changes and the nonadditive litter-mixture effect on litter decomposition (17). Our transplanting decomposer community experiment showed that microbial decomposer communities originating from treatments of litter mixture displayed a higher decomposing ability than those originating from the monospecific tomato root litter treatment, which supported our first hypothesis. These findings thus provided evidence that changes in decomposer communities is one of the underlying mechanisms driving the litter-mixture effect (1, 8, 14). Our results were also commensurate with previous studies showing the functional dissimilarities (e.g., carbon mineralization) among microbial decomposer communities with contrasting compositions (20, 43, 44).

Generally, more diverse communities can provide higher levels of ecosystem functioning (45, 46). Increasing microbial diversity can promote decomposition of organic materials through both facilitative interactions and resource partitioning among microbial species (27, 47). We found that microbial α-diversity was positively related to the decomposition rate of tomato root litter, indicating increased diversity of microbial community might contribute to the enhanced decomposition of tomato root litter in the litter mixtures. Several top-ranking mass loss-predicative OTUs were stimulated by litter mixing such as those belonging to bacterial genera Bacillus, Flavobacterium, Microbacterium, and Pseudoxanthomonas, and fungal genera Fusarium and Cephaliophora, some species of which were reported to have litter-decomposing abilities (37, 46, 48–51). Therefore, stimulation of specific microbial taxa was another possible contributor to the nonadditive litter-mixture effect on decomposition.

### Putative keystone taxa acted as the mediator of the function of microbial decomposer community.

Recent studies have provided evidence for the existence of keystone taxa and highlighted their importance for microbiome assembly and functioning (32–34, 52, 53). For example, the organic material decomposition had strong positive association with certain putative keystone taxa (3, 38). In this study, we found that members of Fusarium and Microbacterium spp. act as putative keystone taxa that mediated the relationship between the nonadditive litter-mixture effect and microbial decomposer community, which supported our second hypothesis. Fusarium sp. fOTU 761 was identified as a putative keystone taxon in the co-occurrence network and the isolated Fusarium sp. F13 showed a strong decomposing ability. These results are in line with the observation that microbial taxa with strong decomposing abilities (e.g., Fusarium sp.) can act as keystone taxa in a decomposer community (3, 38). fOTU 761 was stimulated by litter mixing, indicating that litter mixing could promote decomposition through stimulating microbial taxa with strong decomposing abilities.

Another putative keystone taxon stimulated by litter mixing was Microbacterium sp. bOTU 6632. Although the isolate Microbacterium sp. B26 had limited decomposing ability in pure culture, it promoted litter decomposition when Fusarium sp. F13 or the resident decomposer community was present. These indicate that microbial taxon with a low decomposing ability may act as a putative keystone taxon but their effects on litter decomposition is dependent on other species, such as Fusarium sp., in the community. This also support the view that certain keystone taxa may be able to selectively affect specific members of the community and thus exert their function (33, 38, 54). It should be noted that there are limitations of identifying putative keystone members of a microbial community only by their topological properties within a network (32, 55). Future works should selectively exclude the putative keystone taxa and then re-inoculate them to verify changes in interspecific interactions and the functioning of the community (53, 55).

Plant litter represents an oligotrophic habitat and the ability to degrade complex recalcitrant compounds (e.g., lignin) is constrained to a relatively narrow group of microorganisms (2, 37). Positive interactions among microbial decomposers are supposed to be necessary to litter decomposition, especially for recalcitrant compounds (2, 35, 37, 56). Exogenously addition of Microbacterium sp. B26 promoted Fusarium sp. F13 and the Fusarium sp. abundance in the resident decomposer community. One possible explanation for this stimulatory effect is that these two microbial species could complement each other by supplying different limiting resources. Previous studies have highlighted the complementary roles of bacteria and fungi in decomposing organic materials (1, 2, 56). For example, bacteria can provide nutrients (e.g., nitrogen and vitamin) to fungi, while fungi provide the carbon source to bacteria (36, 56). Another possible explanation is that B26 was able to remove breakdown products of the fungus and thus upregulate the enzyme activity of the fungus (2). Nevertheless, interspecific interactions within a microbial community are highly complex, thus studies using metagenomic and metatranscriptomic approaches are needed to further unravel how putative keystone taxa function within the community.

## CONCLUSIONS

Litter mixing generated the nonadditive litter-mixture effect on litter decomposition through altering microbial decomposer communities. Furthermore, increased relative abundances of some putative keystone taxa (e.g., Fusarium and Microbacterium spp.) promoted the generation of the litter-mixture effect, thus highlighting the important role of putative keystone taxa in mediating the assembly and function of microbial decomposer communities. Putative keystone taxa with limited decomposing activity, such as Microbacterium sp., may indirectly promote litter decomposition through regulating other taxa with strong decomposing activity, such as Fusarium sp.

## MATERIALS AND METHODS

### Soil sampling and preparation of plant litter.

The soil used in this study was collected in July 2019 from an agricultural field in Northeast Agricultural University, Harbin, China (45°41′ N, 126°37′ E). Fifty soil cores (10 cm diameter) were taken from the upper soil layer (0–15 cm) and pooled. Soils were sieved (2 mm), and large stones and plant debris were removed. Then, soils were homogenized, and brought to 60% water holding capacity (WHC), and pre-incubated at 25°C for 5 days before use. The soil was sandy loam, containing soil organic matter, 66.62 g/kg; inorganic nitrogen (ammonium and nitrate), 80.17 mg/kg; Olsen phosphorus, 86.29 mg/kg; available potassium, 125.78 mg/kg; electrical conductivity (1:2.5, wt/vol), 0.31 mS/cm; and pH (1:2.5, wt/vol), 7.43.

Tomato (cv. Baier1628), cucumber (cv. Shengfeng706), and eggplant (cv. Heijin) seedlings with two leaves were transplanted into the field with each crop grown in monoculture in April 2019. After harvest of fruits, roots of these plant species were collected by excavating the soil to a depth of 40 cm in July 2019. These roots were manually separated from the soil under running tap water over a sieve (1 mm mesh). Then, fine roots (diameter < 2 mm) with no sign of senescence were picked up (4). Seeds of maize (cv. Denghai605), wheat (cv. D123), and wild rocket (cv. Shuangji) were directly seeded into the soil in the field as monocultures. Then, 40 days later, the whole plants of maize, wheat and wild rocket were harvested at the vegetative growth stage, which was common for these three cover crops in agricultural production. The collected material of each plant species was mixed and oven-dried at 60°C. Then, litter was chopped into length of 1–2 cm and stored in the dark at room temperature before use. The initial litter chemistry was analyzed for each plant species (see supplemental material).

### Litterbag experiment.

It is difficult to sort component species according to their morphological differences from the litter mixture. Therefore, the two-compartment litterbag method was used estimate the litter‐mixing effects on decomposition of tomato root litter as previously described (40). Nylon litterbags (6 cm × 9 cm) used here contained two compartments separated by a single mesh partition (Fig. S1 in the supplemental material). The upper and bottom sides of the litterbags had 250 μm mesh, which permit entry of small-sized decomposer communities including microorganisms and fauna; while the partition had 1 mm mesh, which permit migrations of decomposer communities between compartments and the contact of litters in different compartments (9, 40). Litterbags containing monospecific litter and all the possible two, four and six-way combinations of tomato with the other five species were included in this experiment (Fig. 1): (i) each single species (i.e., tomato, cucumber, eggplant, maize, wheat, and wild rocket; six types), (ii) each two species combination containing tomato (five types), (iii) each four species combination containing tomato (10 types), and (iv) all six species (one type). Consequently, this experiment encompassed 22 treatments with four levels of litter species richness (i.e., 1, 2, 4, and 6 species). Each litterbag contained a total of 1.8 g of air-dried litter. All mixtures contained equal mass of each component species in a litterbag. For the monospecific treatment, 0.9 g of each species litter was filled in both compartments of the litterbag. For other treatments, tomato root litter was filled in one compartment of the litterbag while other species in the other compartment.

Litterbags were incubated in microcosms (500-mL Kilner jars) containing 300 g fresh field soils and were buried horizontally 5-cm below the soil surface. There was one litterbag per microcosm. Microcosms were sealed with Parafilm and maintained at 25°C in the dark in a growth chamber. Soil moisture content was maintained at 60% WHC. There were nine microcosms for each treatment containing tomato; three of these litter bags were used to measure litter mass loss, another three were served as inocula in the transplanting decomposer community experiment and to isolate culturable bacteria and fungi, and the other three were used to analyze microbial communities. While there were three microcosms for each treatment not containing tomato (monospecific cucumber, eggplant, maize, wheat, and wild rocket), which were used to measure litter mass loss.

Litterbags were harvested after 30 days of incubation. After removed from the microcosms, litter in each litterbag was processed as following: (i) for measuring litter mass loss, each litterbag was opened and soil particles were carefully removed from the samples by washing with tap water over a sieve (200 μm mesh) to ensure that all the litter was retained; litter dry mass was then measured after oven-drying at 60°C to constant weight; (ii) for the transplanting decomposer community experiment and analyzing microbial communities, tomato root litter from each treatment containing tomato were carefully cleaned with a fine brush to remove adhesive soil; one portion of these freshly sampled litter was used as inocula of decomposer communities and for isolating culturable bacteria and fungi, and the other portion was stored at −80°C for DNA extraction.

### Transplanting decomposer community experiment.

The method of transplanting decomposer inoculum to sterilized litter was used to evaluate the effect of changes in decomposer communities on the decomposition of tomato root litter as previously described (43, 44). Briefly, decomposing tomato root litter from the litterbag experiment were used as inocula of decomposer communities (Fig. 1). Undecomposed tomato root litter was milled (2 mm mesh) and sterilized by autoclaving twice in succession and again 24 h later (121°C, 20 min). The absence of culturable microorganisms in sterilized litter was confirmed by adding litter in liquid Luria-Bertani medium. Microcosms (50-mL plastic centrifuge tubes) were constructed by adding 0.1 g of inoculum to 2 g of autoclaved undecomposed litter. The mixture was adjusted to and maintained at 60% WHC. After vortexing, microcosms were sealed with Parafilm and maintained at 25°C in the dark. There were 17 treatments in total: autocalved tomato root litter inoculated with decomposer communities of (i) monospecific tomato (one type), (ii) each two species combination containing tomato (five types), (iii) each four species combination containing tomato (10 types), and (iv) all six species (one type). Each treatment was replicated three times. After 30 days of incubation, litter was harvested, and the dry weight was measured as described above.

### DNA extraction and real-time PCR analysis.

Genomic DNA was extracted from 0.25 g of tomato root litter from the litterbag experiment with the Power Soil DNA isolation kit (MO BIO Laboratories, Carlsbad, USA) following the manufacturer's instructions. The quality of extracted DNA was checked with electrophoresis in a 1.2% (wt/vol) agarose gel and a NanoDrop 2000 spectrophotometer (ThermoFisher Scientific, Wilmington, DE, USA).

Microbial abundances on tomato root litter were assessed by real-time PCR assays conducted with a qTOWER 3G touch real-time PCR system (Analytik Jena, Jena, Germany). The V3 region of the bacterial 16S rRNA gene and the internal transcribed spacer (ITS) regions of the fungal rRNA gene were amplified with primers F338/R518 (57) and ITS1F/ITS4R (58), respectively (detailed PCR conditions are described in the supplemental material).

### Amplicon sequencing and data processing.

The compositions of bacterial and fungal communities on tomato root litter were analyzed with high-throughput sequencing. The V4-V5 regions of the bacterial 16S rRNA gene and the ITS1 region of fungal rRNA gene were amplified with primers F515/R907 (59) and ITS1F/ITS2R (58, 60) with specific overhang Illumina adapters, respectively, as described before (61, 62). Three technically replicated PCRs were performed for each DNA sample (63). To avoid DNA contaminations originating from kits and reagents, sterile water was used as a negative control. The products of the triplicate PCRs were pooled and purified (detailed PCR conditions are described in Supplementary Methods). A second eight-cycle PCR was performed to add dual index and Illumina sequencing adapters using a Nextera XT Index Kit (Illumina Inc., San Diego, CA, USA). Then, PCR products were purified, quantified and normalized prior to pooling. Finally, the DNA library pool was paired-end sequenced (2 × 300) on an Illumina Miseq platform (Illumina Inc.).

Raw sequence reads were processed using the QIIME pipeline (http://qiime.org/). Briefly, adaptor sequence, barcode and 30 low-quality bases at the end of each read were removed. Paired reads were joined (minimum overlapping read length of 20 bp) and quality filtered (Phred score of 20) and reads with less than 200 bp were removed. Chimeras were removed with USEARCH, and sequences were then assigned to OTUs at 97% similarity level using UPARSE (http://drive5.com/uparse/). A representative sequence of each OTU was taxonomically classified using the SILVA 132 (https://www.arb-silva.de/) and Unite 8.0 (http://unite.ut.ee) databases for bacteria and fungi, respectively.

### Isolation and characterization of putative keystone taxa.

Bacteria and fungi strains were isolated from the mixture of decomposing tomato root litter of all treatments in the litterbag experiment, and the taxonomic classification of these strains were identified by sequencing of the 16S rRNA gene and ITS sequences, respectively (detailed methods are described in Supplementary Methods). *In vitro* decomposition tests were performed to assess the decomposing activities of two selected isolates, named B26 and F13, in pure culture, and in mixtures. Each microcosm consisted of a 50-mL plastic centrifuge tube containing 2 g of autoclaved undecomposed tomato root litter. Microcosms were inoculated with different microbial suspensions at a total density of 1 × 10^5^ cell/mL. For the dual mixture, the ratio of each strain was 1:1. Each treatment was replicated five times. Microcosms were sealed with Parafilm and incubated at 25°C in the dark. Fifteen days later, litter was harvested, and the dry weight was measured as described above.

The effect of addition of B26 and F13 on the decomposing ability of the resident microbial community on tomato root litter was evaluated in a microcosm experiment. Briefly, 20 litterbags containing 1.8 g of tomato root litter were prepared as in the litterbag experiment. Tomato root litter were harvested after 30 days of incubation and used as an inoculum of microbial decomposer community. Then, microcosms (50-mL plastic centrifuge tubes) containing 2 g of milled (2 mm mesh) and autoclaved undecomposed tomato root litter were added with each of the following inocula (i) 0.1 g of decomposing tomato root litter, (ii) 0.1 g of decomposing tomato root litter and 500 μL of B26 suspension (1 × 10^4^ cell/mL), (iii) 0.1 g of decomposing tomato root litter and 500 μL of F13 suspension (1 × 10^4^ conidia/mL), and (iv) 0.1 g of decomposing tomato root litter, 250 μL of B26 suspension (1 × 10^4^ cell/mL) and 250 μL of F13 suspension (1 × 10^4^ conidia/mL). Therefore, there were four treatments in this experiment. Each treatment was replicated 10 times. The mixture was adjusted to and maintained at 60% WHC. After vortexing, microcosms were sealed with Parafilm and maintained at 25°C in the dark. Litter was harvested after 30 days of incubation. Half of these samples were used for measuring dry weight, and the other half were stored at −80°C for DNA extraction and quantifying the Fusarium sp. abundance with real-time PCR targeting the translation elongation factor 1 alpha gene with primers Alfie1/Alfie2 (64) (detailed PCR conditions are described in the supplemental material).

### Statistical analyses.

Statistical analyses were conducted in “R” (v4.1.0, http://www.r-project.org/). Relative mass loss of plant litter was calculated as the difference between the initial dry weight and dry weight at harvest, divided by the initial dry weight. All data were checked for normality (Shapiro-Wilk’s test) and homogeneity of variances (Levene’s test). ANOVA was performed to test (i) litter diversity (decomposed into litter species richness and litter species composition) and (ii) litter species identity (i.e., the presence/absence of each species) with litter species richness as a covariate, on litter mass loss and microbial variables. Comparison between two groups was performed using Welch’s *t* test. For more than two groups, means were compared between treatments by the Tukey’s HSD test. Relationships between litter species richness and decomposition rate and microbial variables were tested using linear or log-linear regressions and the one explaining more of the variation was chosen (65).

To avoid potential bias caused by sequencing depth, sequence counts of all samples were normalized to the minimum number of sequence (17,281 16S rRNA gene and 31,985 ITS sequences) per sample. Microbial community α-diversity was calculated as the Shannon index. The β-diversity was analyzed using PCoA based on the Bray-Curtis dissimilarities. PERMANOVA analysis was used to test the effects of litter diversity on community dissimilarity. Microbial OTUs stimulated by litter mixing were identified using the indicator species analysis and likelihood ratio test with the Benjamini-Hochberg (BH) *P* value correction, respectively. Random Forest analysis was conducted to identify microbial OTUs that were predictive of tomato root litter mass loss using the “randomForest” package (66) with 315 random seeds and 1,000 trees. Tenfold cross-validation with five repeats was used to estimate the optimal number of top-ranking OTUs correlated to the decomposition rate using the *rfcv* function in the “randomForest” package (66). The importance of each OTUs was measured using the increase in mean squared error.

To evaluate potential interspecific interactions among microbial taxa, co-occurrence network analysis was performed. Spearman correlations between OTUs with occurrence in more than 20% of samples were calculated. A correlation coefficient was considered statistically robust if Spearman correlation coefficient was > 0.6 and the BH-adjusted *P* value was < 0.01. Some key topological features of the networks (including number of nodes and edges, average connectivity, average path length, clustering coefficient, network density and modularity) and nodes (including node degree and betweenness centrality) were calculated using the “igraph” package (67). OTUs with high node degree and low betweenness centrality values (within the lowest 5% of betweenness centrality and top 5% of node degree values) were considered as possible keystone OTUs (32).

### Data availability.

The raw sequencing data were deposited in the Sequence Read Archive at NCBI with the accession numbers PRJNA739528 and PRJNA739530.
