# Supplementary material for: Litter Mixing Alters Microbial Decomposer Community to Accelerate Tomato Root Litter Decomposition
Source: Microbiol Spectr. 2022 May 23;10(3):e00186-22. doi: 10.1128/spectrum.00186-22 (PMC9241821; doi:10.1128/spectrum.00186-22)
Supplement: SUPPLEMENTAL FILE 1 — Supplemental material. Download spectrum.00186-22-s001.pdf, PDF file, 5.5 MB [file spectrum.00186-22-s001.pdf]

SUPPORTING INFORMATION

Litter Mixing Alters Microbial Decomposer Community to Accelerate Tomato Root Litter Decomposition

Xue Jin,[a,b] Zhilin Wang,[b] Fengzhi Wu,[a,b] Xiaogang Li,[c] Xingang Zhou[a,b]

[a] Key Laboratory of Biology and Genetic Improvement of Horticultural Crops (Northeast Region), Ministry of Agriculture and Rural Affairs, Northeast Agricultural University, Harbin 150030, China.

[b] Department of Horticulture, Northeast Agricultural University, Harbin 150030, China

[c] College of Biology and the Environment, Nanjing Forestry University, Nanjing 210037, China

Correspondence: Xingang Zhou, E-mail: xgzhou@neau.edu.cn.

SUPPLEMENTARY METHODS

Litter chemical analysis. The collected material of each litter species was milled (2 mm mesh). Total nitrogen concentration was measured by Kjeldahl distillation after digesting the plant material with sulfuric acid (1). Total phosphorus concentration was determined colorimetrically using the molybdenum blue method after digesting the plant material with sulfuric acid and hydrogen peroxide (2). The calcium concentration was evaluated by the complexometric titration method (3). Total carbon content was measured with a FlashSmart™ elemental analyzer (ThermoFisher Scientific, Waltham, USA). Lignin content was determined gravimetrically using the downscaled acid-detergent fiber procedure (1).

Real-time PCR analyses. Real-time PCR assays were performed in a 25 μl reaction mixture containing 12.5 μl of 2×Real SYBR Mixture (Tiangen Biotech, Beijing, China), 0.2 μM of each of the forward and reverse primers, 5 ng of DNA, and sterile deionized water was used to bring the total volume to 25 μl. The PCR protocols were 94°C for 5 min; followed by 94°C for 45 s, 56°C for 45 s for bacteria (57.5°C for 45 s for fungi, 50°C for 40 s for *Fusarium* sp.), 72°C for 60 s, 30 cycles; and a final extension at 72°C for 10 min. Standard curves were created with 10-fold dilution series of plasmids containing the target gene. The threshold cycle values obtained for each sample were compared with the standard curve to calculate the copy number of the target gene. Sterile water was used as a negative control to replace the template. The efficiency of the reaction was between 97% and 100% (based on the slopes of the standard curves). All amplifications were performed in triplicate.

PCR conditions for Illumina MiSeq sequencing. PCR was performed in a reaction mixture of 25 μl consisting of 12.5 μl of 2×Taq PCR MasterMix (Tiangen Biotech, Beijing, China), 0.2 μM of each of the forward and reverse primers, 5 ng of DNA; sterile deionized water was used to bring the

total volume to 25 μl. The PCR was performed with an EasyCycler PCR System (Analytik Jena, Jena, Germany) using a program of 95°C for 5 min; followed by 35 cycles of 95°C for 30 s, 55°C for 30 s, 72°C for 45 s; and a final extension at 72°C for 10 min. To avoid DNA contaminations originating from kits and reagents, sterile water was used as a negative control.

Isolation and identification of bacteria and fungi from decomposing tomato root litter. Bacteria and fungi on decomposing tomato root litter were isolated as describe before. (4-6). Briefly, decomposing tomato root litter from different treatments were mixed. Then, 2 g of litter sample was pulverized using a Waring blender (JJ-2A, Changzhou, China) and washed with 10 ml of sterile distilled water. Serial dilutions (10^{-1} to 10^{-7}) were prepared and plated onto Petri dishes (9 cm diameter) containing the following medium: 1/5 tryptic soy agar medium (5 g/L tryptone, 1.65 g/L soy peptone, 1.65 g/L NaCl and 15 g/L agar) for isolation of fast-growing bacteria, low-nutrient agar medium (5 mg/L $MgSO_4$ $7H_2O$, 5 mg/L KNO_3, 13 mg/L K_2HPO_4 $3H_2O$, 0.6 mg/L $CaNO_3$ $4H_2O$, 25 mg/L glucose, 2 mg/L enzymatic casein hydrolysate and 13 g/L agar) for isolation of slow-growing bacteria, malt extract agar medium (20 g/L malt extract and 20 g/L agar) for isolation of fast-growing fungi, and Miura's medium (1 g/L glucose, 1 g/L KH_2PO_4, 0.2 g/L $MgSO_4 \cdot 7H_2O$, 0.2 g/L KCl, 2 g/L $NaNO_3$, 0.2 g/L yeast extract and 13 g/L agar) for isolation of slow-growing fungi. Moreover, tryptic soy agar and low-nutrient agar medium were supplemented with 30 mg/L cycloheximide, and malt extract agar and Miura's medium were supplemented with 30 μg/L streptomycin. Plates were incubated at 25°C in the dark and observed daily. Seven replicate Petri dishes were prepared for each dilution and for each medium. Any bacterial colony and fungal hypha or spore appearing on the plates were transferred to freshly prepared yeast extract peptone dextrose agar plates and malt extract agar plates, respectively, for purification and identification, respectively. Bacteria and fungi isolations were

finally selected from the 10^{-3} and 10^{-6} dilutions, respectively. In total, 375 bacterial strains and 215 fungal strains were isolated. Based on feasibility of handling in culture, 222 bacterial and 127 fungal strains with different morphology were selected and identified using DNA sequencing.

Genomic DNA was extracted from liquid bacterial cultures or fungal mycelium using the Power Soil DNA Isolation Kit (MO BIO Laboratories, Carlsbad, USA) following the manufacturer's instructions. Bacterial 16S rRNA gene and the ITS regions of the fungal rRNA gene were amplified with primers 27F/1492R (7) and ITS1F/ITS4R (8), respectively. PCR assays were performed in a 25 μl reaction mixture containing 12.5 μl of 2×Real SYBR Mixture (Tiangen Biotech, Beijing, China), 0.2 μM of each of the forward and reverse primers, 2.5 ng of DNA, and sterile deionized water was used to bring the total volume to 25 μl. The PCR protocols were 94°C for 5 min; followed by 94°C for 30 s, 58°C for 45 s, 72°C for 90 s for bacteria (60 s for fungi), 30 cycles; and a final extension at 72°C for 10 min. The amplicons were purified and commercially sequenced by Sangon Biotech Co., Ltd., Shanghai, China. Then, the obtained sequences were compared to other sequences on the BLAST database at the National Center for Biotechnology Information.

We obtained 11 *Microbacterium* and 59 *Fusarium* sp. isolates and isolates belonging to other genus were discarded. After elimination of potential clonal duplicates, *i.e.*, isolates with 100% identity of the 16S rRNA gene or ITS sequences (9), we obtained 10 *Pseudomonas* sp. isolates. Neighbor-joining trees for these bacteria and fungi were generated, respectively, using MEGA (v10.17) (bootstrap values based on 1,000 resampling) (10). The 16S rRNA gene or ITS sequences of the microbial isolates were matched with keystone OTUs in the co-occurrence network. The sequences of bacterial 16S rRNA gene and the ITS regions of the fungal rRNA gene were trimmed at the sites of primer sets F515/R907 and ITS1F/ITS2R (8, 11, 12), respectively. Form these isolates, we selected bacterial isolate B26 and fungal isolate F13, which displayed 100% sequence similarity with bacterial

OTU6632 and fungal OTU761, respectively.

SUPPLEMENTARY FIGURES

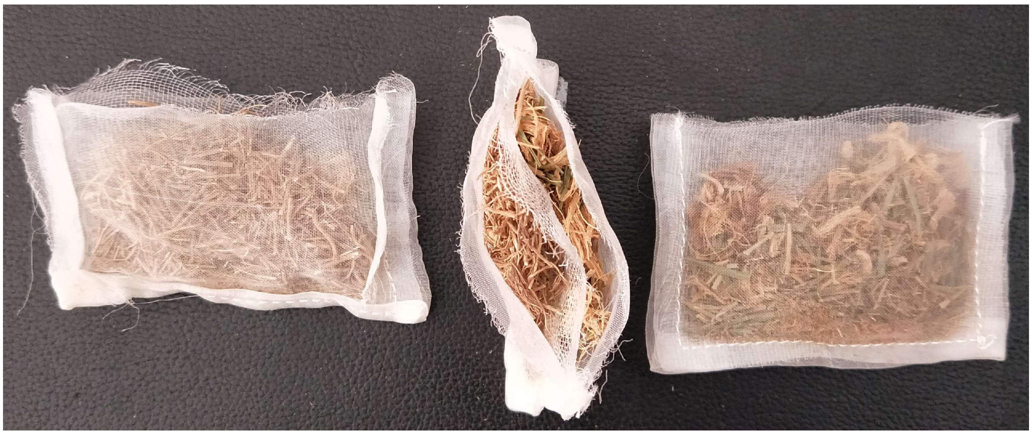

FIG S1 Representative photographs of the two-compartment litterbags used in this study. Nylon litterbags (6 cm × 9 cm) used contained two compartments separated by a single mesh partition. The upper and bottom sides of the litterbags had 250 µm mesh, while the partition had 1 mm mesh.

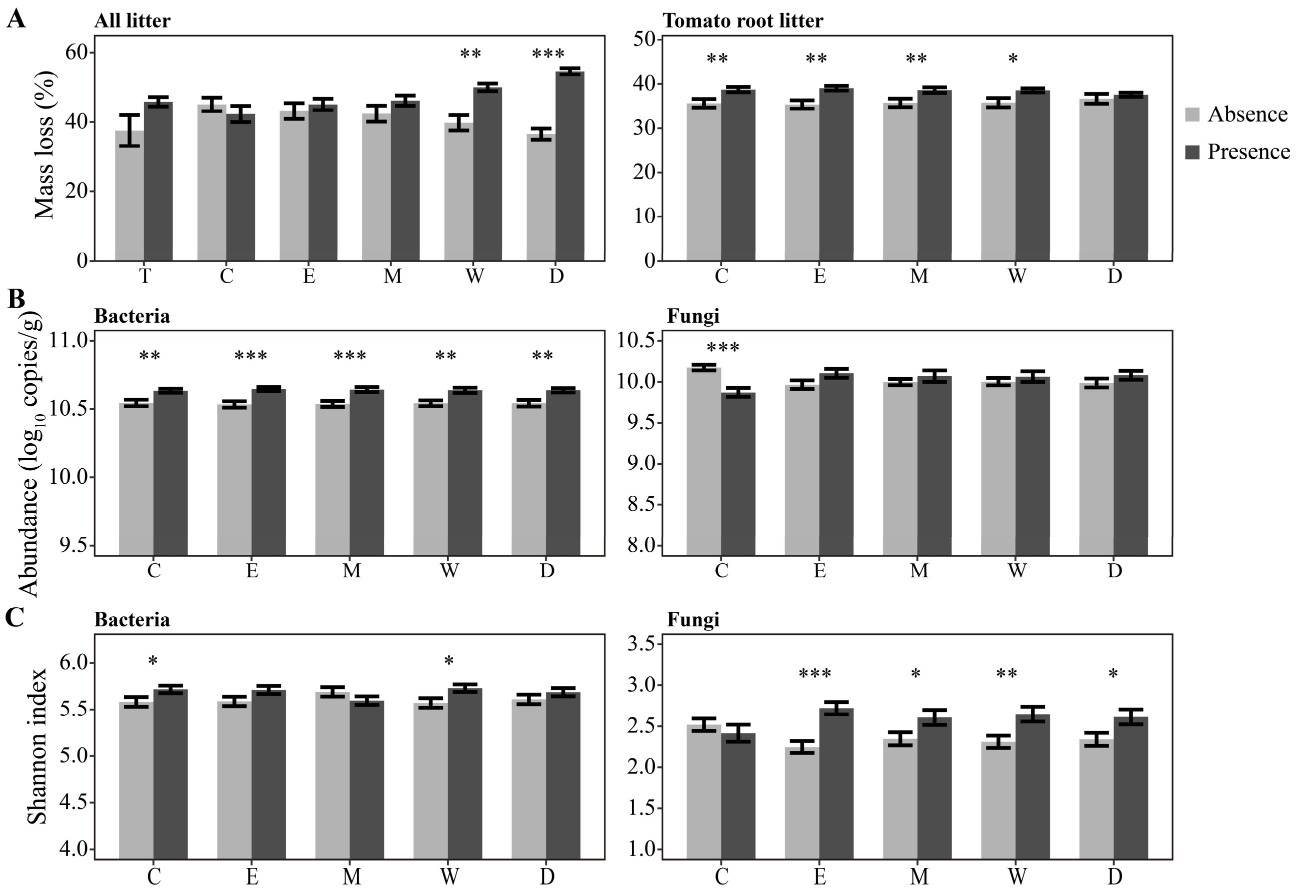

FIG S2 Effects of the presence of each litter species on litter mass loss, and microbial abundances and α-diversities. (A) Effects of the presence of each litter species on mass loss of the all litter, and other litter species on mass loss of tomato root litter. (B) Effects of the presence of other litter species on the abundance of bacterial and fungal communities on tomato root litter. (C) Effects of the presence of other litter species on the Shannon indices of bacterial and fungal communities on tomato root litter. *, ** and *** indicate significant effects of certain species presence at $P < 0.05$, 0.01 and 0.001, respectively, as estimated by ANOVA with litter species richness as a covariate.

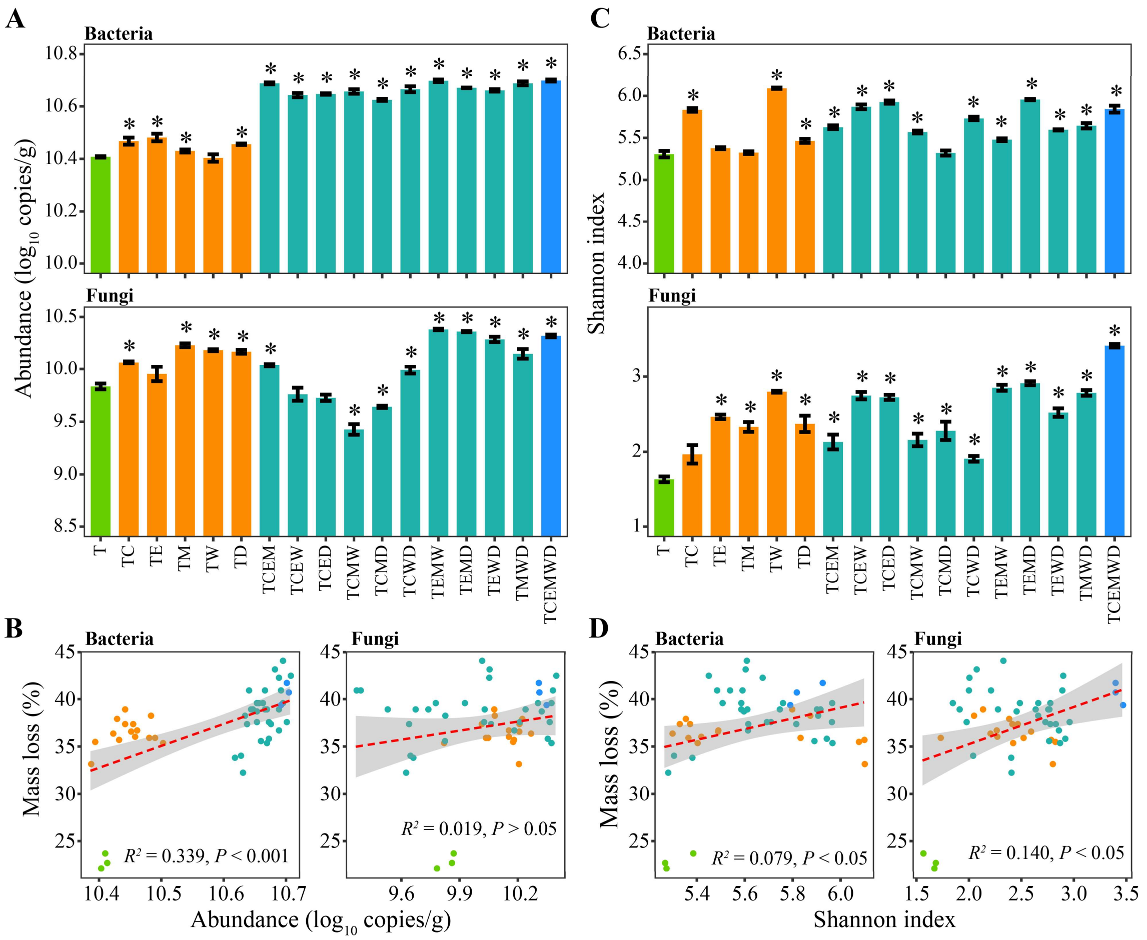

FIG S3 Microbial abundance and diversity for each treatment and their relationship with tomato root litter mass loss. (A) Bacterial and fungal abundance on tomato root litter for each treatment. (B) Relationships between microbial abundances and tomato root litter mass loss. (C) Shannon indices of bacterial and fungal communities on tomato root litter for each treatment. (D) Relationships between Shannon indices and tomato root litter mass loss. For (A) and (C), values are represented as mean ± SE (n=3). * indicate significant different with the monospecific treatment (Student's t-test, P < 0.05). T, tomato; C, cucumber; E, eggplant; M, maize; W, wheat; D, wild rocket. Dashed red lines show the linear regression fitting and shaded areas represent 95% confidence intervals.

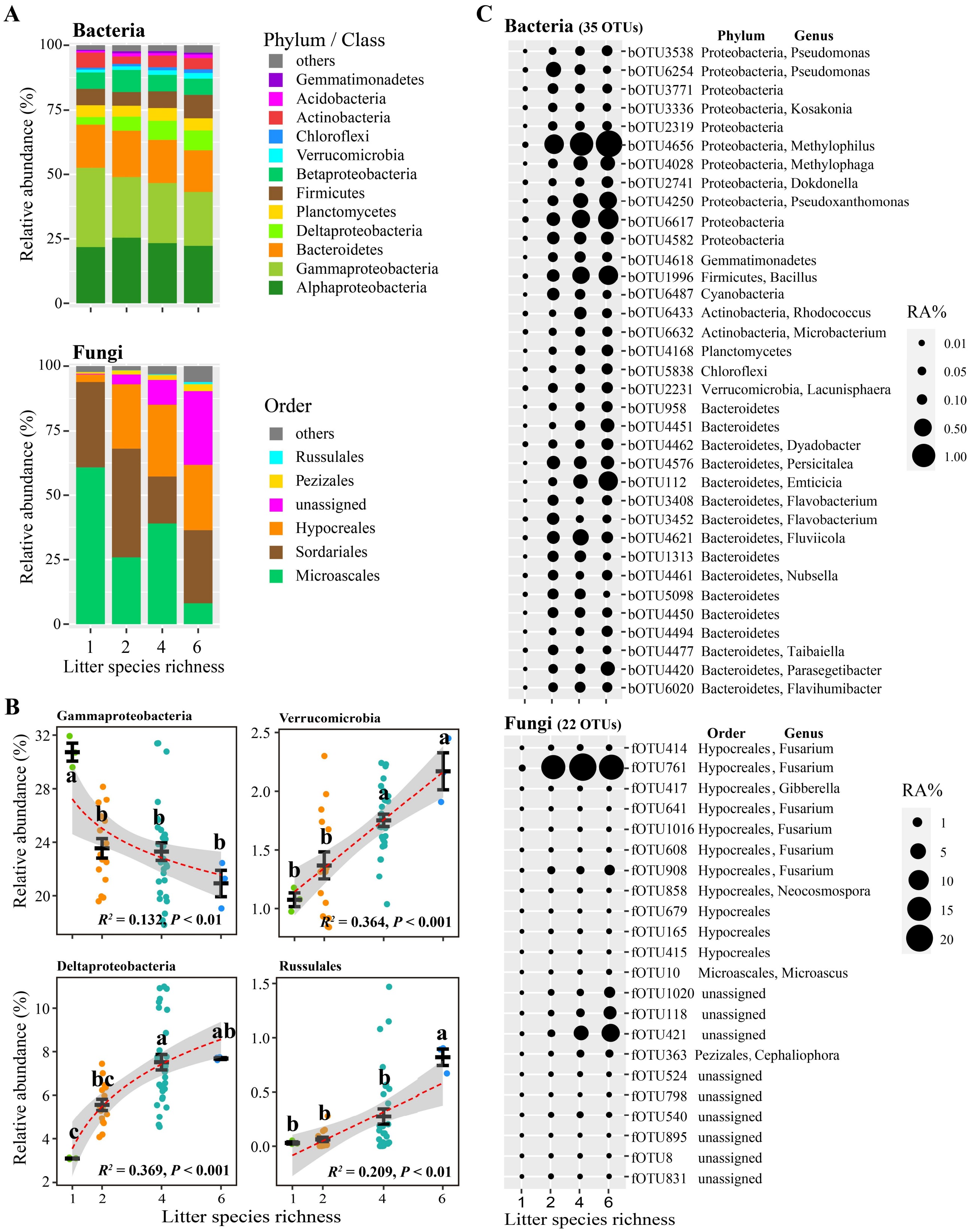

FIG S4 Microbial taxa sensitive to litter mixing. (A) Relative abundances of main bacterial phyla/*Proteobacteria* classes (mean relative abundances > 1%) and fungal order (mean relative abundances > 0.5%) of each litter species richness. T, tomato; C, cucumber; E, eggplant; M, maize;

W, wheat; D, wild rocket. (B) Effects of litter species richness on relative abundances of main bacterial phyla/*Proteobacteria* classes and fungal orders. Only taxa had significant linear relationships with litter species richness were shown. Different letters indicate significant differences (Tukey's HSD test, $P < 0.05$). Dashed red lines show the linear or log-linear regression fittings and shaded areas represent 95% confidence intervals. (C) Phylogenetic information of bacterial and fungal OTUs stimulated by litter mixing as determined by both indicator species analysis and likelihood ratio test.

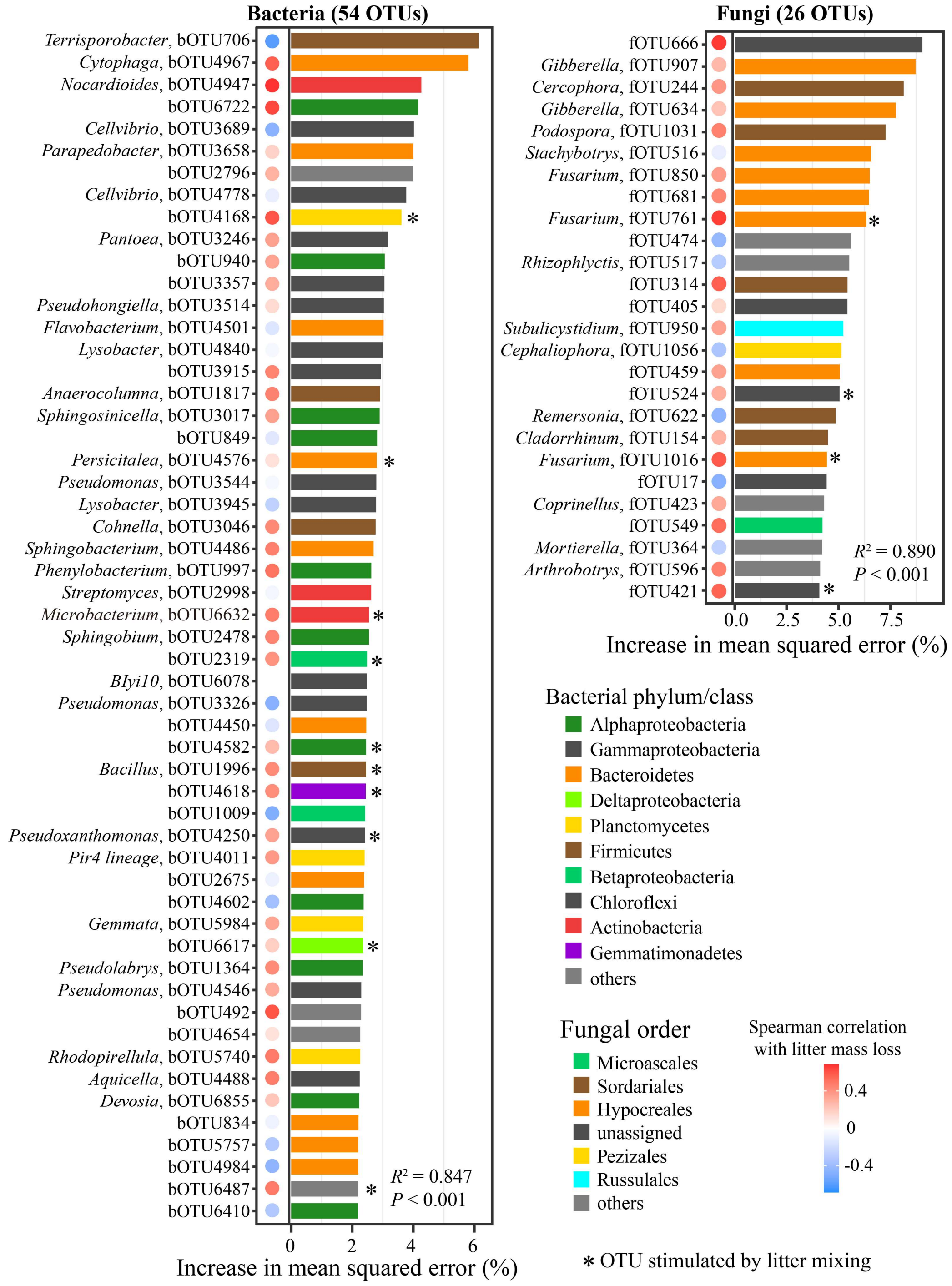

FIG S5 Regression Random Forest models detected OTUs that are predictive of mass loss of tomato root litter. The bubbles on the left show the Spearman's correlations between the relative abundance of each OTU and tomato root litter mass loss. The middle bar plots show the importance value of each OTU estimated by Regression Random Forest models. The optimal number of top-ranking OTUs correlated to the decomposition rate was estimated using tenfold cross-validation with five repeats. Taxa that could be assigned to the genus level were shown as genus, otherwise were shown

as OTU id.

SUPPLEMENTARY TABLES

TABLE S1 Litter traits of the six litter species used in the experiment

	Carbon (mg/g)	Nitrogen (mg/g)	Phosphorus (mg/g)	Calcium (μg/g)	Lignin (mg/g)
Tomato	451.67 ± 3.63 a [a]	14.72 ± 0.54 d	3.35 ± 0.19 d	2.53 ± 0.15 b	263.70 ± 7.11 a
Cucumber	435.54 ± 1.73 b	15.73 ± 0.12 cd	5.97 ± 0.07 b	2.15 ± 0.16 b	187.32 ± 4.86 b
Eggplant	452.89 ± 4.48 a	16.39 ± 0.32 c	3.18 ± 0.09 de	2.44 ± 0.12 b	259.33 ± 4.27 a
Maize	401.81 ± 2.47 c	18.42 ± 0.22 b	2.89 ± 0.11 e	1.40 ± 0.04 c	81.63 ± 2.92 d
Wheat	436.81 ± 4.42 b	19.23 ± 0.33 b	6.64 ± 0.09 a	2.32 ± 0.10 b	133.80 ± 5.10 c
Wild rocket	396.33 ± 4.28 c	25.32 ± 0.74 a	5.51 ± 0.14 c	5.88 ± 0.08 a	63.70 ± 2.17 e

[a] Values are mean ± SE (n=6). Different letters indicate significant differences (Tukey's HSD test, $P < 0.05$).

TABLE S2 Key topological features of the empirical and Erdös-Rényi random networks

Empirical networks						Random networks		
N[a]	E	AC	APL	ACC	MD	APL	ACC	MD
811	7058	17.41	3.50	0.44	0.41	2.66	0.02	0.20

[a] N – number of nodes; E – number of edges; PE – number of positive edges; NE – number of negative edges; AC – Average connectivity; APL – Average path length; ACC – Average clustering coefficient; MD – modularity. Numbers in the brackets indicate percentages of negative edges (%).

Supplementary references

1. Graça MAS, Bärlocher F, Gessner MO. 2005. Methods to study litter decomposition: a practical guide. Springer Science & Business Media, The Netherlands.

2. Grimshaw H, Allen S, Parkinson J. 1989. Nutrient elements. Blackwell Scientific Publications, Oxford.

3. Garcia-Palacios P, Milla R, Delgado-Baquerizo M, Martin-Robles N, Alvaro-Sanchez M, Wall DH. 2013. Side-effects of plant domestication: ecosystem impacts of changes in litter quality. New Phytol 198:504-513. https://doi.org/10.1111/nph.12127

4. Semenov AM, van Bruggen AHC, Zelenev VV. 1999. Moving waves of bacterial populations and total organic carbon along roots of wheat. Mol Ecol 37:116-128. https://doi.org/10.1007/s002489900136

5. Cornejo FH, Varela A, Wrigh SJ. 1994. Tropical forest litter decomposition under seasonal drought: nutrient release, fungi and bacteria. Oikos 70:183-190. https://doi.org/10.2307/3545629

6. Osono T. 2005. Colonization and succession of fungi during decomposition of *Swida controversa* leaf litter. Mycologia 97:589-597. https://doi.org/10.3852/mycologia.97.3.589

7. Heuer H, Krsek M, Baker P, Smalla K, Wellington EM. 1997. Analysis of actinomycete communities by specific amplification of genes encoding 16S rRNA and gel-electrophoretic separation in denaturing gradients. Appl Environ Microbiol 63:3233-3241. https://doi.org/10.1128/aem.63.8.3233-3241.1997

8. Gardes M, Bruns TD. 1993. ITS primers with enhanced specificity for basidiomycetes: application to the identification of mycorrhiza and rusts. Mol Ecol 2:113-118. https://doi.org/10.1111/j.1365-294X.1993.tb00005.x

9. Durán P, Thiergart T, Garrido-Oter R, Agler M, Kemen E, Schulze-Lefert P, Hacquard S. 2018.

Microbial interkingdom interactions in roots promote *Arabidopsis* survival. Cell 175:973-983. https://doi.org/10.1016/j.cell.2018.10.020

10. Tamura K, Peterson D, Peterson N, Stecher G, Nei M, Kumar S. 2011. MEGA5: molecular evolutionary genetics analysis using maximum likelihood, evolutionary distance, and maximum parsimony methods. Mol Biol Evol 28:2731-2739. https://doi.org/10.1093/molbev/msr121

11. Zhou J, Wu L, Deng Y, Zhi X, Jiang Y-H, Tu Q, Xie J, Van Nostrand JD, He Z, Yang Y. 2011. Reproducibility and quantitation of amplicon sequencing-based detection. ISME J 5:1303-1313. https://doi.org/10.1038/ismej.2011.11

12. Bokulich NA, Mills DA. 2013. Improved selection of internal transcribed spacer-specific primers enables quantitative, ultra-high-throughput profiling of fungal communities. Appl Environ Microbiol 79:2519-2526. https://doi.org/10.1128/aem.03870-12
